# Kinetical Study, Thermo-Mechanical Characteristics and Recyclability of Epoxidized Camelina Oil Cured with Antagonist Structure (Aliphatic/Aromatic) or Functionality (Acid/Amine) Hardeners

**DOI:** 10.3390/polym13152503

**Published:** 2021-07-29

**Authors:** Chiara Di Mauro, Aratz Genua, Alice Mija

**Affiliations:** 1 Institut de Chimie de Nice, UMR CNRS 7272, Université Côte d’Azur, 06108 Nice, France; chiara.di-mauro@univ-cotedazur.fr; 2 CIDETEC, Basque Research and Technology Alliance (BRTA), Paseo Miramon 196, 20014 Donostia-San Sebastian, Spain; agenua@cidetec.es

**Keywords:** dual-crosslinked networks, dual-dynamic hardeners, recyclability, epoxidized camelina oil, mechanical recycling, chemical recycling

## Abstract

In an attempt to prepare sustainable epoxy thermosets, this study introduces for the first time the idea to use antagonist structures (aromatic/aliphatic) or functionalities (acid/amine) as hardeners to produce reprocessable resins based on epoxidized camelina oil (ECMO). Two kinds of mixtures were tested: one combines aromatic/aliphatic dicarboxylic acids: 2,2′-dithiodibenzoic acid (DTBA) and 3,3′-dithiodipropionic acid (DTDA); another is the combination of two aromatic structures with acid/amine functionality: DTBA and 4-aminophenyl disulfide (4-AFD). DSC and FT-IR analyses were used as methods to analyze the curing reaction of ECMO with the hardeners. It was found that the thermosets obtained with the dual crosslinked mechanism needed reduced curing temperatures and reprocessing protocols compared to the individual crosslinked thermosets. Thanks to the contribution of disulfide bonds in the network topology, the obtained thermosets showed recycling ability. The final thermomechanical properties of the virgin and mechanical reprocessed materials were analyzed by DMA and TGA. The obtained thermosets range from elastomeric to rigid materials. As an example, the ECMO/DTBA_70_4-AFD_30_ virgin or reprocessed thermosets have tan *δ* values reaching 82–83 °C. The study also investigates the chemical recycling and the solvent resistance of these vitrimer-like materials.

## 1. Introduction

The interest in and development of dynamic covalent chemistry (DCvD) [1] and reactions that use dynamic covalent bonds have grown exponentially in recent years. The introduction of interchangeable and reversible bonds in polymer networks, due to the sensitivity to external stimuli, is playing a fundamental role in the emerging technologies of self-repairing or recycling systems. The opportunity of reconnecting chemical bonds allows the reduction in waste in landfills and prolonging the life cycle of products according to the circular economy and global warming.

The transesterification reaction, in which an ester and an alcohol are in equilibrium with a different ester/alcohol pair, is an example of a direct exchange reaction. This reaction has long been described by Leibler et al. [2,3], preparing vitrimers starting from DGEBA and a mix of di- and tricarboxylic acids, for which the exchange reactions are thermally activated.

There are several potentially dynamic covalent functions, including acetals, borazaromatic anhydrides, borate esters, disulfides, hydrazones, imines and olefins [1,4,5]. Among the first reactions used in DCvC, the disulfide exchange reaction was one of the foremost studied reactions. The metathesis of aromatic disulfides and the exchange capacity are known to occur at room temperature [6,7]. Rekondo et al. [8] synthesized a polyurethane elastomer using an aromatic diamine disulfide crosslinker, 4-aminophenyl disulfide (4-AFD), and showed that self-healing occurred at room temperature with a repairing efficiency of more than 95%. Zeng et al. [9] obtained a reprocessable castor oil-based polyurethane by incorporating 4-AFD and reported [10,11,12,13,14] that the mechanical properties were completely recovered after reprocessing. For the first time, our team produced reprocessable, repairable and recyclable epoxidized vegetable oil (EVO) thermosets by using 2,2′-dithiodibenzoic acid (DTBA) as a crosslinker. The synthesized networks showed reprocessable and repairable abilities for up to 10 cycles. However, diamine hardeners have also been used in the literature to produce epoxy thermosets starting from bio-based monomers [15,16,17].

Recently, Chen et al. [18] synthetized a poly (esteramide) vitrimer from castor oil via melt condensation with sebacic acid, polyamide 1010 monomer salt and 4-AFD, obtaining a reprocessable material, several times, without the erosion of network structures or of mechanical properties. Liu et al. [19] reported a catalyst-free epoxy vitrimer based on epoxidized soybean oil (ESO)/4-AFD that showed comparable mechanical properties, gel fraction and chemical structure after repeatedly cutting and compression molding it several times.

The combination of epoxy matrices has long been used, specifically EVOs with DGEBA, with the advantage of being transparent and better for the environment than 100% petroleum-based epoxy resins [20,21,22]. However, very few studies have been published regarding the mix of hardeners. Ding et al. [23] prepared bio-based thermosets by curing ELO with adipic acid/glutaric anhydride catalyzed by N, N-4-dimethylaminopyridine (DMAP), with the properties being modulated depending on the ratio of the two hardeners. Williams et al. [24] synthesized vitrimers from DGEBA with citric acid (CA)/sebacic acid (SA) by adding a small amount of n-alkylamine. The authors highlighted that the presence of tertiary amines catalyzes the transesterification reaction by increasing the physical crosslinks and promoting the associations of alkyl chains.

In this regard, our team developed reversible double-crosslinking networks by curing epoxidized linseed oil (ELO) with different amounts of two diacid disulfide hardeners: DTBA/3,3′-dithiodipropionic acid (DTDA) initiated by imidazole, with the disulfide exchange mechanism being proven by high-performance liquid chromatography coupled with mass spectroscopy [25].

**Figure 1:**
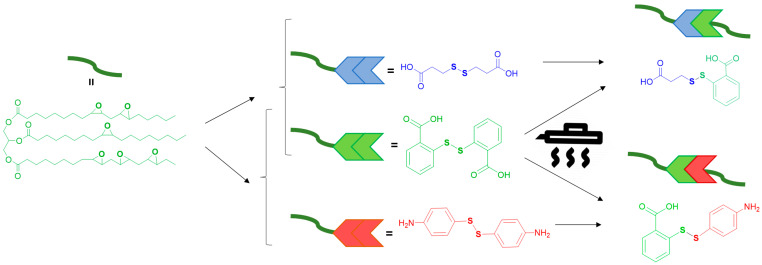
Schematized connectivity structures between ECMO with aliphatic–aromatic diacids or diacid–diamine crosslinkers.

In this study, we obtained a series of thermosets by curing epoxidized camelina oil (ECMO) with different ratios of aromatic/aliphatic diacids DTBA_x_DTDA_y_ and in parallel with an acid/amine DTBA_x_ 4-AFD_y_ mixture of hardeners, to modulate the properties of the final epoxy resins (Figure 1). The aim of this work was to analyze the structure–property correlation, i.e., the effect of the chemical structure of the disulfide hardener regarding its reactivity with the ECMO, but especially the networks’ recyclability. For this reason, we designed two kinds of hardener systems: (i) one full aromatic (DTBA_x_ 4-AFD_y_) where we compared acid vs. amine dynamic hardeners, i.e., the electron-withdrawing group vs. electron-donating one and (ii) one where the aliphatic vs. aromatic structures were analyzed regarding their impact on thermosets’ properties and, again, especially their recycling. Matxain et al. [26] reported theoretical calculations for a series of aliphatic and aromatic disulfide compounds and so evaluated the electronic conditions related to the radical-mediated mechanism of self-healing. The authors showed that bond dissociation energy (BDE) values for dialkyl disulfides are around 65 kcal/mol and only 50 kcal/mol in diaryl disulfides. Moreover, they showed that phenyl rings substituted with nucleophiles such as NH_2_ have reduced BDE by delocalization of the electron of the generated sulfenyl radical into the aromatic ring. Therefore, starting with these theoretical computations, we aimed here to investigate how the substitutions of the disulfide hardeners affect not only the crosslinking kinetics but also the thermosets’ overall properties, especially their recycling. To the best of our knowledge, this study introduces for the first time the idea to use antagonist structures (aromatic/aliphatic) or functionalities (acid/amine) as hardeners to produce reprocessable EVO resins. Modulating the percentages of one or the other hardener in the preparation of thermosetting will guarantee targeted properties according to the specific application required. The reactivity study of these new formulations was carried out using differential scanning calorimetry (DSC) and Fourier transform infrared spectroscopy (FT-IR). The thermo-mechanical properties of virgin and recycled materials were analyzed by thermogravimetric (TGA) and dynamic mechanical analyses (DMA). Finally, the solvent stability vs. recycling ability were evaluated to predict a potential recyclability scenario from a chemical point of view.

## 2. Materials and Methods

### 2.1. Materials

Epoxidized camelina oil (ECMO) was purchased from Specific Polymers. The chemical structure and characteristics are reported in Table S1. The hardeners and the initiator are commercially available and were purchased from Sigma-Aldrich (Merck): 2,2′-dithiodibenzoic acid (DTBA), 95%, 4-aminophenyl disulphide (4-AFD), 98%, 3,3′-dithiodipropionic acid (DTDA), 99%, and imidazole (IM), 99%. All reagents were used without further purification.

### 2.2. Preparation of Crosslinked Networks

The epoxy thermosets were prepared using a stoichiometric ratio R 1:1 for epoxy/acid (e/a) and epoxy/amine functionalities, with the epoxy group considered monofunctional during the reaction with an acid [27] and bifunctional with an amine [28]. 

A total of five epoxy/DTBA_x_DTDA_y_ formulations were prepared, in which the origin of dicarboxylic acid groups was: 100% DTBA (ECMO/DTBA_100_), 70% DTBA–30% DTDA (ECMO/DTBA_70_DTDA_30_), 50% DTBA–50% DTDA (ECMO/DTBA_50_DTDA_50_), 30% DTBA–70% DTDA (ECMO/DTBA_30_DTDA_70_) and 100% DTDA (ECMO/DTDA_100_). A second series of five epoxy/acid/amino formulations was also prepared: 70% DTBA–30% 4-AFD (ECMO/DTBA_70_4-AFD_30_), 50% DTBA–50% 4-AFD (ECMO/DTBA_50_4-AFD_50_), 30% DTBA–70% 4-AFD (ECMO/DTBA_30_4-AFD_70_) and 100% 4-AFD (ECMO/4-AFD_100_).

The composition of the formulations and their acronyms are given in Table S2.

The preparation of resins based on ECMO with DTBA_100_, DTDA_100_ and DTBA_x_DTDA_y_ was carried out according with a procedure described elsewhere. The ECMO formulations with the DTBA_x_4AFD_y_ mixture were prepared by melting the aromatic diamine in the epoxy monomer and consequently added DTBA and the initiator. 

For the DMA analysis, the specimens were prepared in special rectangular molds. The analyzed cured resins and recycled samples had rectangular dimensions of 30 × 7 × 2 mm^3^ (length × width × thickness).

### 2.3. Mechanical Reprocessing Procedure

For the mechanical reprocessing, a piece of crosslinked thermoset was ground, and the obtained small pieces were compressed between two Kapton films in a CARVER heating press. The applied reprocessing conditions (temperature, time and pressure) are reported in Table S5.

### 2.4. Chemical Recycling Procedure

The complete dissolution of the thermosets was tested in a solution of 5 wt.% of 1–4 dithiothreitol in DMF, for 24 h at 50 °C. A greener recycling of thermosets was performed by immersing the samples in 1N NaOH for 72 h at 80 °C. The tests were conducted on rectangular 10 × 10 × 2 mm^3^ specimens.

### 2.5. Methods

Differential scanning calorimetry (DSC) experiments were carried out on a Mettler-Toledo DSC 3 apparatus controlled by STAR Software developed by Mettler-Toledo. Freshly prepared mixtures of about 5–7 mg were placed into 40 µL aluminum crucibles, and the copolymerization reactions were thermally conducted by heating the crucibles at 10 °C/min in the range 25–250 °C. DSC was used also to evaluate the *T*_g_ of the cured thermosets by applying two cycles of heating–cooling (at 10 °C·min^−1^) in the range from −60 °C to 180 °C.

The FT-IR analyses were recorded using a Thermo Scientific Nicolet iS50 FT-IR spectrometer with a deuterated L-alanine-doped triglycine sulfate (DLaTGS) detector in attenuated total reflectance (ATR) mode. The absorption bands were recorded in the range of 4000–525 cm^−1^ by applying 32 scans and a resolution of 4 cm^−1^. The data were analyzed using OMNIC software.

Thermogravimetric analysis (TGA) measurements were carried out on a Mettler-Toledo TGA 2. Samples of about 10 mg were placed into 70 µL alumina pans. The cured networks were heated at 10 °C·min^−1^ from 25 to 1000 °C under 50 mL·min^−1^ air.

Dynamic mechanical analyses (DMAs) were performed by using a Mettler-Toledo DMA 1 instrument, equipped with STAR software. The analyzed samples had rectangular dimensions of 30 × 7 × 2 mm^3^ (length × width × thickness) and the analyses were carried out with the tension method. Elastic moduli (*E*′) and damping factors (tan *δ*) were collected at a 3 °C·min^−1^ heating rate from −50 to 150 °C and 1.0 Hz frequency. Crosslinking density was calculated by Flory’s expression, as shown by Equation (1):

(1) $$\nu =\frac{E^{\prime }}{3RT}\;$$

where *E*’ is the storage modulus of the thermoset in the rubbery plateau region at *T*_g_ + 50 °C, *R* is the gas constant and *T* is the absolute temperature in Kelvin. 

Solvent resistance experiments were carried out by immersing the samples for 72 h at room temperature in ethanol, acetone, THF and 1 N NaOH solution. The tests were conducted on rectangular 10 × 10 × 2 mm^3^ specimens.

## 3. Results and Discussion

### 3.1. Reactivity Studies on the Function of the Dual-Dynamic Crosslinker Combination

The reactivity of ECMO/DTBA_x_DTDA_y_ formulations was studied by dynamic DSC and the obtained results are displayed in Figure S1 and the data are listed in Table S3. We can firstly notice that the systems epoxy/DTBA_100_ and epoxy/DTDA_100_ show a single exothermic peak, attributed to the epoxy–acid curing reactions (Figure S1A). In the thermograms of ECMO/DTBA_x_DTDA_y_, we can observe that when decreasing the DTBA percentage, the *T*_on_ of crosslinking reactions decreases from 137 to 126 °C for ECMO/DTBA_70_DTDA_30_ and, thereafter, it increases again, to 141 °C for ECMO/DTDA_100._ The same effect can be observed for the *T*_peak_ that moves from 155 °C for the ECMO/DTBA_100_ curing formulation to 143 °C for the ECMO/DTBA_30_DTDA_70_ system and increases again to 147 °C for ECMO/DTDA_100_. A reduction in the reaction enthalpy can be observed in the presence of the aliphatic hardener. A lower Δ*H* was measured for ECMO/DTBA_50_DTDA_50_ and in the meantime a higher secondary reaction was observed. Ding et al. [23], using ELO cured with different amounts of glutaric anhydride (GA) and adipic acid (AA), observed that with the increase in AA content, the Δ*H* increases, with the mixture GA_60_AA_40_ at ~286 J·g^−1^ and being less reactive than the system 20:80 with an enthalpy of ~302 J·g^−1^.

All the blends with a mixture of diacid crosslinkers showed a faster *α* conversion compared with the blends made with individual DTBA or DTDA (Figure S1B), and ECMO/DTBA_30_DTDA_70_ exhibits a faster conversion, in good correlation with the highest enthalpy of the system.

**Figure 2:**
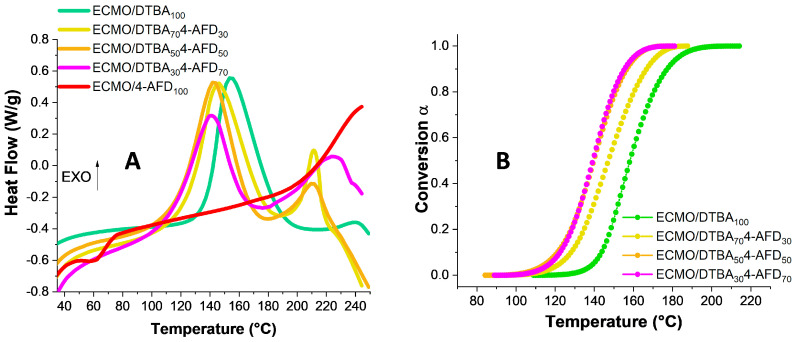
(**A**) DSC thermograms and (**B**) degree of conversion α for the ECMO/DTBA_x_4-AFD_y_ hardeners.

When the ECMO was reacted with 4-AFD diamine crosslinker (Figure 2A), the exotherm was shifted to high temperatures with a *T*_peak_ at ~248 °C. When the reaction occurred in the presence of an acid/amine mixture of crosslinkers, all the thermograms were shifted to lower temperatures. By adding the 4-AFD in the DTBA blends, a subsequent reduction in reaction enthalpy was observed, from 171 J·g^−1^ for the ECMO/DTBA_100_ system to 106 J·g^−1^ for the blend ECMO/DTBA_30_ 4-AFD_70_. This result can be correlated with the hydrogen bonds in the networks containing the mixture of hardeners. However, by blending DTBA_x_4-AFD_y_, the shape of the thermograms became more complex with the appearance of a second broad exothermic peak at high temperatures around 215–230 °C. The second exothermic peak increases in the presence of diamine in the blends. This peak can be attributed to the epoxy homopolymerization which takes place because of the stoichiometric imbalance produced by the hindrance and the high functionality in both hardener acids as well as amine in the mixture. It should be noted that the enthalpy of ECMO/DTBA_x_4-AFD_y_ reactions, given in Table 1, can correspond to several processes, such as epoxy–acid, epoxy–amine and acid–amine reactions. 

Finally, the full conversion was reached faster when the diamine dynamic crosslinker was added, with comparable values for ECMO/DTBA_50_4-AFD_50_ and ECMO/DTBA_30_4-AFD_70_ systems (Figure 2B).

### 3.2. Structural Evolution by FT-IR Studies 

**Figure 3:**
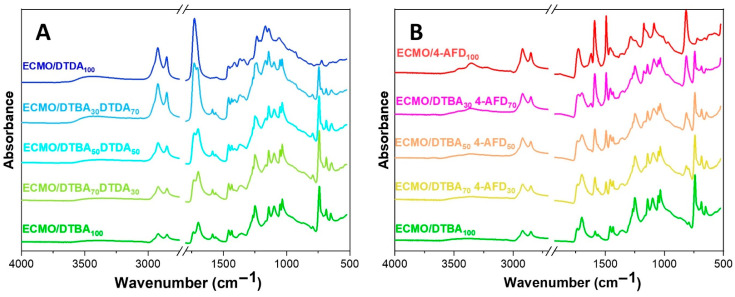
FT-IR spectra of the reactive groups in the uncured mixtures of ECMO/DTBA_x_DTDA_y_ (**A**) and ECMO/DTBA_x_4-AFD_y_ (**B**).

The FT-IR spectral absorbances as a function of the formulations of the crosslinking ECMO/DTBA_x_DTDA_y_ and ECMO/DTBA_x_4-AFD_y_ mixtures are illustrated in Figure 3. Increasing the percentage of DTDA, the peak due to the C=O_ester_ is shifted from 1737 cm^−1^ in ECMO/DTBA_100_ to 1729 cm^−1^ in ECMO/DTBA_30_DTDA_70_, up to the formation of a single band at 1728 cm^−1^ in the fully DTDA blend (Figure S2).

Similarly, Figure 3B and Figure S3 show the characteristic bands for ECMO/DTBA_x_4-AFD_y_ uncured mixtures. The comparison of FT-IR spectra of the uncured mixture and cured ECMO/4-AFD_100_ resin is given in Figure S4; we can observe the stretching of the oxirane ring at 3030 cm^−1^ (located from 3050 and 2990 cm^−1^) and the disappearance of the epoxy groups (~1000 cm^−1^) in the cured sample. The stretching of the primary amine in the uncured mixture is located at 3460 and 3364 cm^−1^ with an additional combination band at ~3213 cm^−1^ in the cured resin attributed to the stretching vibration of the formed amide groups. The end of the curing protocol was also confirmed by the formation of the secondary amine (in the region 3450–3300 cm^−1^), the formation of stretching and the combination band of –N^+^–H at ~3000 cm^−1^ and the absence of the epoxy group absorbance.

**Figure 4:**
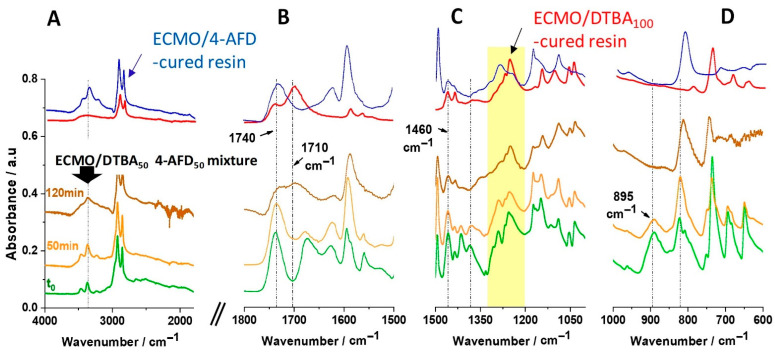
Evolution FT-IR spectra of the ECMO/DTBA_50_ 4-AFD_50_ mixture during curing at 130 °C for different curing times (t = 0, 50, 90, 120 min) in comparison with cured resin of ECMO/DTBA_100_ and ECMO/4-AFD_100_: (**A**) zoomed in view of the region from 4000-1800 cm^−1^; (**B**) zoomed in view of the region from 1800–1500 cm^−1^; (**C**) zoomed in view from 1500 to 1000 cm^−1^ and (**D**) zoomed in view from 1000 to 600 cm^−1^.

Figure 4 illustrates the FT-IR comparison of the reactive ECMO/DTBA_50_ 4-AFD_50_ mixture and of the final ECMO/4-AFD_50_ and ECMO/DTBA_100_ thermosets. Due to excessive overlapping of the 4-AFD signal with the epoxy peak in the region from 850 cm^−1^ to 800 cm^−1^, it was not possible to study its conversion over time for ECMO/DTBA_x_4-AFD_y_ systems. However, the FT-IR spectra allowed us to follow the evolution of the most relevant reactive groups and the formation of crosslinked networks during the isothermal curing at 130 °C taking, as an example, the formulation ECMO/DTBA_50_4-AFD_50_. 

During the first hour of the reaction, the decrease in the broad peaks at 1678 cm^−1^ and 895 cm^−1^, assigned to free carboxylic groups, together with the simultaneous appearance of a new ester “C=O” absorption band at about ~1740 cm^−1^ and 1710 cm^−1^, are noticed. It indicates the occurrence of an esterification addition reaction between acid–epoxy, confirmed by the presence of the same peak in the cured ECMO/DTBA_100_ resin (Figure 4). 

Concerning the evolution of amine signals of 4-AFD, the quantification of primary and secondary amines in the epoxy/amine reactive system is limited because of their positions in the FT-IR spectra. The band of N-H stretching of primary amines is very close to the strong O-H as well as to the secondary amine absorption bands in the 3600–3300 cm^−1^ region. However, regarding the FT-IR spectrum of the cured ECMO/4-AFD resin and comparing it with that of ECMO/DTBA_50_ 4-AFD_50_ after 2 h of curing at 130 °C, it seems that the epoxy–amine reaction also occurred. We can notice in these systems the presence of –OH stretching vibration at 3463 cm^−1^ (Figure 4 and Figure S4) and a new peak also appearing at 3354 cm^−1^, corresponding to –NH stretching vibration. These bands are proof of hydrogen non-covalent interactions in ECMO/DTBA_x_ 4-AFD_y_ systems.

### 3.3. Thermoset and Recycled Material Characterization 

The combination of DTDA and 4-AFD with the DTBA allowed us to obtain hardeners that reacted with ECMO at lower temperatures compared with the individual hardeners. Therefore, these combinations permitted us to decrease the curing and post-curing temperatures. Once the curing process was optimized by the help of several DSC investigations to check the full completion of the crosslinking reaction, the thermoset samples were characterized (their thermal and chemical stability, mechanical and dynamic mechanical properties). Table S4 displays the characteristics of the cured thermosets and the curing and post-curing protocol for the selected systems. 

The presence of exchangeable S-S bonds combined with transesterification reactions gives the materials excellent reprocessing properties. The recycling conditions and characteristics of the reprocessed thermosets are displayed in Table S5. The synergy of the reversible mechanisms combined the hardeners association gave lower recyclable conditions than those of thermosets obtained with individual crosslinker.

#### 3.3.1. Glass Transition Evaluation by DSC and DMA Analyses

**Figure 5:**
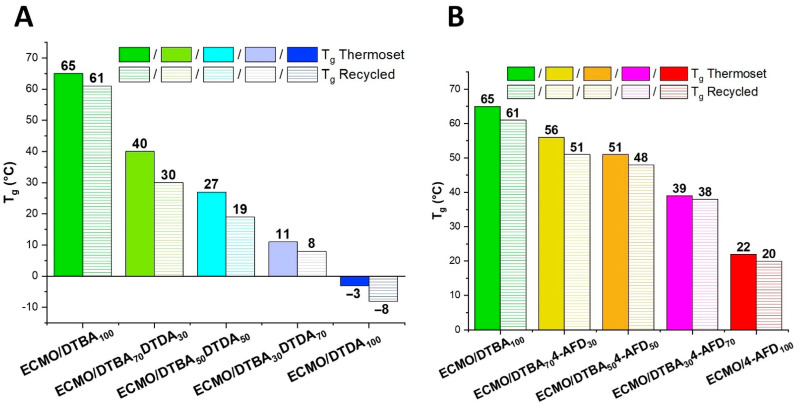
*T*_g_ for the virgin and recycled ECMO/DTBA_x_DTDA_y_ (**A**) and ECMO/DTBA_x_4-AFD_y_ (**B**) thermosets.

The glass transitions of virgin and reprocessed thermosets were determined by DSC. The effects of the hardeners’ combination and their proportions are shown in Figure 5. The ECMO/DTBA_100_ thermoset presents a higher *T*_g_ (~65 °C) due to the aromatic contribution to the network’s rigidity. Interestingly, the *T*_g_ of ECMO/4-AFD_100_ is three times lower, ~22 °C, probably because the lower reactivity of this system produces a deficient crosslink density. Increasing the aliphatic contribution by DTDA percentage, the glass transitions values move to a lower value, reaching −3 °C for ECMO/DTDA_100_, a sign of a very soft and elastic thermoset. A slow decrease in the *T*_g_ range was observed after the recycling process.

**Figure 6:**
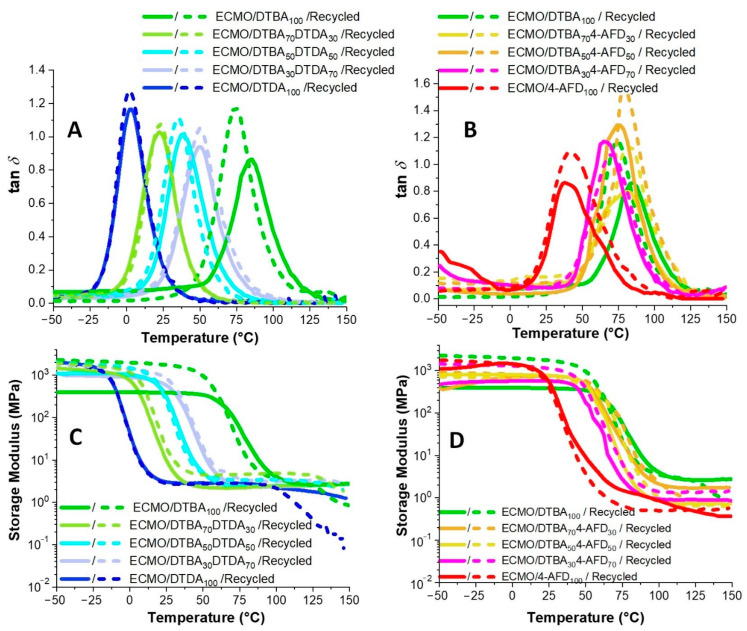
DMA curves of tan δ and storage moduli vs. temperature for the virgin or recycled thermosets based on ECMO cured with: (**A**) and (**C**) DTBA_x_DTDA_y_; (**B**) and (**D**) DTBA_x_4-AFD_y_.

The dynamic mechanical properties of both ECMO/DTBA_x_DTDA_y_ and ECMO/DTBA_x_4-AFD_y_ virgin and reprocessed samples were studied by DMA. Figure 6 shows the variation in storage moduli (*E*’) and tan *δ* values as a function of the temperature. The corresponding data are summarized in Table 2. The loss moduli variations for the different compositions vs. temperature are displayed in Figure S5. 

The maximum value of the tan *δ* peak was taken as the α-transition temperature. As already observed in the DSC results, the tan *δ* increases gradually with the DTBA content in the hardener mixtures due to the improved stiffness of the network structure: from 3 °C for ECMO/DTDA_100_ to 85 °C for ECMO/DTBA_100_, while the thermoset made with 4-AFD has a value of ~52 °C for ECMO/4-AFD_100_.

Liu et al. [19] reported, for ESO/4-AFD thermosets, a damping factor from 26 to 34 °C as a function of the applied curing protocol, while the crosslink density varies from 0.095 to 0.190 mmol·cm^−3^. 

The reprocessed ECMO/DTBA_50_DTDA_50_ thermosets exhibit a smaller decrease in tan *δ* than that of recycled ECMO/DTBA_100_; in contrast, an increase in the damping factor was observed for DTBA_x_4-AFD_y_.

Figure 6C,D show the variation in the storage moduli with the temperature for the systems with acid-type hardeners, DTBA_x_DTDA_y_ (Figure 6C) or combined acid/amine ones, DTBA_x_4-AFD_y_ (Figure 6D). In Figure 6C, there are four domains: the glassy region, the transition region, the rubbery plateau and the terminal or viscoelastic region. Therefore, reaching *T* >> *T*_g_, it is possible to observe the passage from the amorphous solid rubbery state to the viscous liquid state in some recycled ECMO/DTBA_x_DTDA_y_ thermosets (Figure 6C), associated with an increased chain mobility. Especially in the recycled ECMO/DTDA100 and ECMO/DTBA100 thermosets, we can notice the presence of liquid–liquid transitions, with *T*_ll_ reported as being correlated with a disintegration, a “quasi-melting”, on heating, of long-lived and stable segments—segments associated with neighboring segments in amorphous polymers, depending on the thermal history and the formation of stable associations of the segments during material cooling [29]. The *T*_ll_ transition corresponds therefore to a short-range order–disorder transition, i.e., a loss of intramolecular ordering. We can hypothesize that in the ECMO/DTBA_x_DTDA_y_ recycled systems, the dual-dynamic mechanism of transesterification and S-S cleavage activated by the heating at *T* >> *T*_g_ favors the occurrence of this *T*_ll_ transition. Moreover, this loss of order appears to be exacerbated in the recycled ECMO/DTDA100 aliphatic thermoset, while for the recycled ECMO/DTBA100 aliphatic–aromatic thermoset, the E’ drop at this transition is lower. This behavior can be correlated with the fact that the aromatic disulfides metathesis is more efficient in terms of exchange of the disulfide bonds. In contrast, this *T*_ll_ transition is not present in the recycled ECMO/DTBA_x_4-AFD_y_ thermosets (Figure 6D). We can attribute this result to the structural contribution of non-covalent interactions, such as as the hydrogen bonds (highlighted by FT-IR spectra from Figure 4 and Figure S4) and π–π stacking (both DTBA and 4-AFD are aromatics), that keep the disulfide bonds closer and allow the constant reconstruction and rearrangement of the network during the heating. In these systems, the non-covalent interactions seem to favor the conformationally regular intramolecular regions.

From the *E*’ plots (Figure 6C,D), it can be observed that the virgin or recycled DTBA_x_DTDA_y_ and DTBA_x_4-AFD_y_ thermosets show higher storage moduli in the glassy state than that of the virgin ECMO/DTBA_100_ and that the recycled thermosets have higher *E*’ values in the glassy region. As can be seen, the epoxy resins cured by DTBA_x_DTDA_y_ show higher storage moduli in the glassy region or in the rubbery plateau, compared with the resins cured by DTBA_x_4-AFD_y_. Moreover, in these latter systems, the recycled samples show increased values of tan *δ*.

#### 3.3.2. Materials’ Thermal Stabilities 

**Figure 7:**
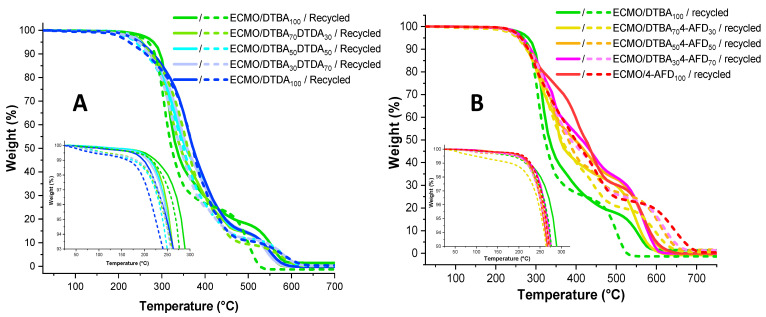
TGA and zoomed-in view of the *T*_5%_ area (from 25–300 °C) for the virgin and recycled ECMO thermosets: DTBA and DTDA combination (**A**) and DTBA and 4-AFD and mixtures (**B**).

Thermogravimetric analyses of ECMO/DTBA_x_DTDA_y_ thermosets show a 5% weight loss (*T*_5%_) range from 245 °C for ECMO/DTDA_100_ to 270 °C for ECMO/DTBA_100_ (Figure 7, and Table 3). The reduced thermal stability of thermosets with a higher ratio of aliphatic crosslinkers can be attributed to the ease of breaking the aliphatic chains with respect to aromatic DTBA and could also be associated with the lower crosslinking density of the network based on DTDA crosslinkers, as shown in Table 2. No significant thermal stability reduction was observed after the recycling protocol, the T5% decreasing with ~5–10 °C, excepting the recycled ECMO/DTDA100 resin, with a higher decrease of T5% with ~20 °C.

The ECMO/DTBA_x_4-AFD_y_ thermosets exhibit closer values of *T*_5%_ (Figure 7), centered at 260 °C, whatever the ratio of the hardeners, showing a strong influence of the diamine crosslinker as obtained for other thermoset resins made with 4-AFD [30,31]. Comparable results were reported by Liu et al. [19] for epoxidized soybean oil cured with 4-AFD; the authors observed excellent thermal stability with an onset decomposition temperature of around 270 °C. Zhou et al. [30] for a vitrimer based on bis (4-glycidyloxyphenyl) disulfide cured with 4-AFD obtained 5% weight loss at 275 °C.

Two well-separated decomposition processes can be observed in the TGA and DTG curves (Figure S6) for the thermosets obtained with DTBA_x_DTDA_y_. The first degradation stage involves pyrolysis with the breakdown of the ester–methylene linkages and of hydroxyls, and the second involves the thermo-oxidative degradation of the products formed in the first step. Concerning the DTBA_x_4-AFD_y_ thermosets, complex DTG thermograms with three-step degradations can be observed in Figure 7. Similar results were found and reported by Liu et al. [19], who attributed the first stage to the thermal decomposition of disulfide bonds, the second to the ESO moieties and the last to the thermo-oxidation of the benzene rings and π–π conjugations.

### 3.4. Solvent Resistance and Chemical Recyclability

**Figure 8:**
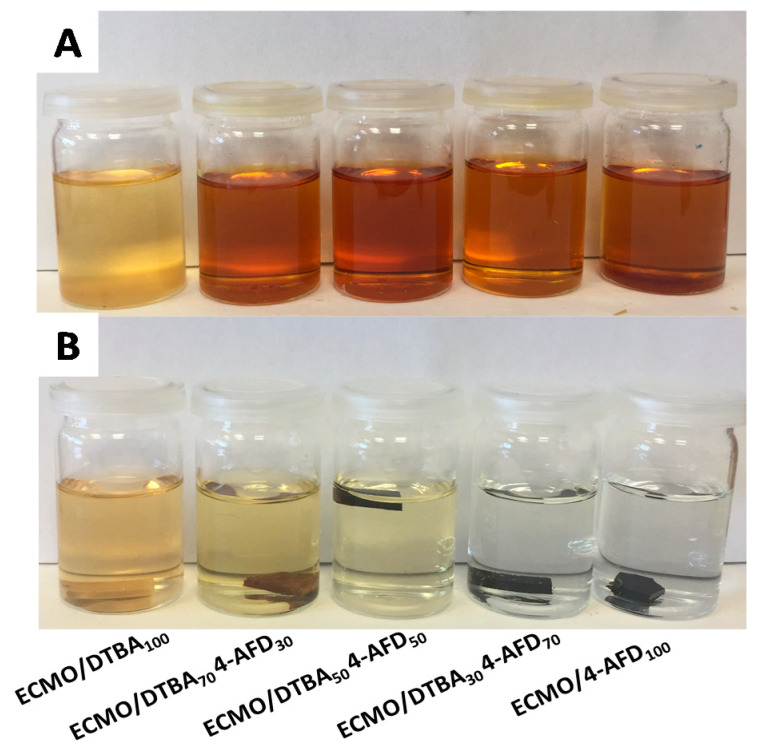
(**A**) Chemical recycling in DMF solution of dithiothreitol for ECMO/DTBA_X_ 4-AFD_X_ after 24 h at 50 °C and (**B**) solvent resistance in 1N NaOH solution after 3 days at room temperature.

The epoxy resins based on disulfide bonds can be recycled in DMF solution of dithiothreitol [14,28,30]. Figure 8A and Figure S8A present the full degradation and chemical recycling ability after 24 h at 50 °C of the thermosets based on ECMO/DTBA_x_DTDA_y_ and ECMO/DTBA_x_ 4-AFD_y_.

The solvent resistance of the thermosetting resins was tested in ethanol, acetone, THF and 1N NaOH for 72 h at room temperature. Figure S8 displays the good resistance of the ECMO/DTBA_x_ 4-AFD_y_ thermosets in ethanol and acetone. However, the solvent resistances of ECMO/DTBA_x_DTDA_y_ resins in 1 N NaOH (Figure S7B) show differences, exhibiting higher solubility than those based on DTBA_x_4-AFD_y_ (Figure 8B) and with an increased ratio of the diamine crosslinker, the thermosets became completely insoluble. Odriozola et al. [32] showed comparable results in 1 N NaOH solution for fiber-reinforced polymer composites based on DGEBA/4-AFD epoxy resin. The same team reported that epoxy composites were insoluble in different chemicals, such as THF, toluene, acetone, ethanol and 1 N HCl. As for ELO/DTBA_100_ thermosets, all the ECMO/DTBA_x_DTDA_y_ resins show full and sustainable recyclability after 3 days at 80 °C (Figure S7B).

Finally, in contrast with the degradation behavior of the reprocessable resins based on ELO and ESO with DTBA, Figure S9 displays the progressive resistance of ECMO/DTBA_x_4-AFD_y_ resins in THF. As vitrimers, these thermosets swell without degradation in “a good solvent” [3,32,33,34]. 

## 4. Conclusions

This study reveals the proof of concept of mixing hardeners with opposite properties, aliphatic–aromatic and acid–amine, and the use of these combinations to cure epoxidized camelina oil, a bio-based epoxy monomer with 5.24 meq/g. All the molecules used as hardeners contain dynamic disulfide bonds, to engender reprocessability and recyclability in the thermosets. Moreover, the mixture of crosslinkers causes a reduction in the temperatures of the reaction, as shown in the DSC thermograms, so there are less drastic conditions for curing and reprocessing, compared with the systems with an individual crosslinker. The addition of DTBA, as an aromatic diacid, in ECMO/DTDA and ECMO/4-AFD matrices, produces an increase in the glass transition, the thermal stability and the crosslink density.

On the one hand, the thermosets exhibited strong solvent resistance while, on the other hand, thanks to the nature of the disulfide bonds in the network structure, full chemical recycling was obtained.

We can use these outcomes to create a series of thermosetting materials by modulating the ratios between the hardeners to obtain a variety of properties, ensuring, at the same time, recycling ability.

## Figures and Tables

**Table 1:** DSC results for the curing of ECMO/DTBA_x_4-AFD_y_ systems.

Thermosets	*T*_peak_ 1 (°C)	Reaction Interval 1	Δ*H*_1_ (J·g^−1^)	*T*_peak_ 2 (°C)	Reaction Interval 2	Δ*H*_2_ (J·g^−1^)
ECMO/DTBA100	171	137–187	155	/	/	/
ECMO/DTBA_70_ 4-AFD_30_	145	125–175	155 ± 1	212	202–217	28 ± 1
ECMO/DTBA_50_ 4-AFD_50_	143	118–166	151 ± 1	211	197–221	14 ± 1
ECMO/DTBA_30_ 4-AFD_70_	141	115–160	106 ± 1	220	214–235	50 ± 1
ECMO/4-AFD_100_	248	155–/	/	/	/	/

**Table 2:** Thermomechanical properties of the virgin and reprocessed resins.

Thermosets	Tan *δ*, Virgin/Recycled (°C)	Tan *δ*_max_, Virgin/Recycled	E’_glassy plateau_, Virgin/Recycled (MPa)	E’_rubbery plateau_, Virgin/Recycled (MPa)	Crosslink Density, Virgin/Recycled (mmol/cm^3^)
ECMO/DTBA_100_	85/75 ± 1	0.9/1.2	400/2290	2.78/0.83	0.71/0.24
ECMO/DTBA_70_DTDA_30_	50/50 ± 1	0.9/1.0	1000/1820	2.47/3.32	0.25/0.34
ECMO/DTBA_50_DTDA_50_	39/35 ± 1	1.0/1.1	1100/2030	2.60/3.10	0.27/0.38
ECMO/DTBA_30_DTDA_70_	22/22 ± 1	1.0/1.1	1500/1970	2.56/4.57	0.27/0.53
ECMO/DTDA_100_	3/2 ± 1	1.2/1.3	2280/2000	2.82/2.87	0.35/0.33
ECMO/DTBA_70_ 4-AFD_30_	82/83 ± 1	0.79/1.1	400/900	1.70/0.87	0.48/0.18
ECMO/DTBA_50_ 4-AFD_50_	74/81 ± 1	1.3/1.53	800/720	0.74/0.66	0.35/0.28
ECMO/DTBA_30_ 4-AFD_70_	65/70 ± 1	1.2/1.07	400/1460	0.91/1.35	0.23/0.25
ECMO/4-AFD_100_	36/41 ± 1	0.86/1.1	1110/1780	0.53/0.51	0.21/0.17

**Table 3:** *T*_5%_ for virgin and recycled thermosets.

Thermosets	*T*_5%_ Virgin/Recycled (°C)	Thermosets	*T*_5%_ Virgin/Recycled (°C)
ECMO/DTBA_100_	270/265 ± 1		
ECMO/DTBA_70_DTDA_30_	255/245 ± 1	ECMO/DTBA_70_ 4-AFD_30_	260/255 ± 1
ECMO/DTBA_50_DTDA_50_	250/240 ± 1	ECMO/DTBA_50_ 4-AFD_50_	260/260 ± 1
ECMO/DTBA_30_DTDA_70_	250/245 ± 1	ECMO/DTBA_30_ 4-AFD_70_	265/260 ± 1
ECMO/DTDA_100_	245/225 ± 1	ECMO/4-AFD_100_	260/260 ± 1

## Supplementary Materials

The following are available online at https://www.mdpi.com/article/10.3390/polym13152503/s1. Figure S1. DSC thermograms for the ELO bio-epoxy monomers combined with the diacid aromatic/aliphatic hardeners (a) and the dynamic diacid–diamine aromatic crosslinkers (b) in R 1:1 and 1 wt% of IM; Figure S2. DSC thermograms and degree of conversion α (B) for the ECMO bio-epoxy monomers combined with the diacid aromatic/aliphatic hardeners in R 1:1 and 1 wt% of IM; Figure S3. FT-IR zoomed-in view of the ECMO/DTBA_x_DTDA_y_ blends in the region from 1800 to 1150 cm^−1^; Figure S4. Evolution of FT-IR spectra of the cured ECMO/DTBA_x_4-AFD_y_ blends: (A) zoomed-in view of the region of 4000–2600 cm^−1^ and (B) 1850–1550 cm^-^^1^; Figure S5. FT-IR evolution of the reactive groups of the ELO/4-AFD_100_ in presence of 1 wt.% IM mixture and after the curing protocol; Figure S6. Loss modulus vs. temperature for the virgin and recycled (A) ECMO/DTBA_x_DTDA_y_ and (B) ECMO/DTBA_x_4-AFD_y_ thermosetting resins; Figure S7. DTG for the neat ECMO/DTBA_x_DTDA_y_ and combination of the diacid crosslinkers (A) and neat ECMO/DTBA_x_4-AFD_y_ and mixture of diacid/diamine (B); Figure S8. (A) Chemical recycling in DMF solution of dithiothreitol for ECMO/DTBA_x_DTDA_x_ after 24 h at 50 °C and (B) solvent resistance in 1N NaOH solution after 3 days at room temperature; Figure S9. Solvent resistance tests in (A) ethanol and (B) acetone after 72 h at room temperature for the ECMO/DTBA_x_4-AFD_y_ resins; Figure S10. Solvent test in THF for ECMO/DTBA_x_4-AFD_y_ resins after 72 h at room temperature; Table S1. Structures and characteristics of the selected reagents; Table S2. Material designation and composition of ECMO/DTBA_x_DTDA_y_–DTBAx 4-AFD_y_ blends; Table S3. DSC results for the ELO/DTBA_x_DTDA_y_ and ELO/DTBA_x_4-AFD_y_ systems; Table S4. DSC results for the ECMO/DTBA_x_DTDA_y_ systems; Table S5. Curing and post-curing protocol for the ECMO/DTBA_x_DTDA_y_ and ECMO/DTBA_x_4AFD_y_ blends; Table S6. Recycling conditions and aspects of the reprocessed ECMO/DTBA_x_DTDA_y_ and ECMO/DTBA_x_4AFD_y_ blends.

## Abbreviations

EVO: Epoxidized vegetable oil; ECMO: Epoxidized camelina oil, DCA: Dicarboxylic acids; DTBA: 2,2′-dithiodibenzoic acid; DTDA: 3,3′-dithiodipropionic acid; 4-AFD: 4-aminophenyl disulfide; IM: Imidazole; FT-IR: Fourier transform infrared spectroscopy; DSC: Differential scanning calorimetry; DMA: Dynamic mechanical analysis; TGA: Thermogravimetric analysis; DTG: Derivative thermogravimetry; vs.: Versus.
