# Supplementary material for: Kinetical Study, Thermo-Mechanical Characteristics and Recyclability of Epoxidized Camelina Oil Cured with Antagonist Structure (Aliphatic/Aromatic) or Functionality (Acid/Amine) Hardeners
Source: Polymers (Basel). 2021 Jul 29;13(15):2503. doi: 10.3390/polym13152503 (PMC8347613; doi:10.3390/polym13152503)
Supplement: Supplementary file 1 [file polymers-13-02503-s001.zip › polymers-1288091-supplementary.pdf]

Article

Kinetical Study, Thermo-Mechanical Characteristics and Recyclability of Epoxidized Camelina Oil Cured with Antagonist Structure (Aliphatic/Aromatic) or Functionality (Acid/Amine) Hardeners

Chiara Di Mauro [1], Aratz Genua [2] and Alice Mija [1,*]

1 Institut de Chimie de Nice, UMR CNRS 7272, Université Côte d'Azur, 06108 Nice, France; chiara.di-mauro@univ-cotedazur.fr
2 CIDETEC, Basque Research and Technology Alliance (BRTA), Paseo Miramon196, 20014 Donostia-San Sebastian, Spain; agenua@cidetec.es
* Correspondence: alice.mija@univ-cotedazur.fr

Supplementary Materials

Table S1. Structure and characteristics of ECMO.

EVOs	Structure	Epoxy content (meq/g)	Mw (g/mol)
ECMO	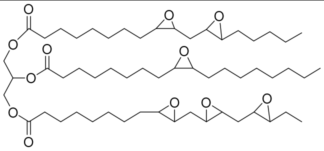 Epoxidized Camelina Oil	5.24	965
		**Mw (g/mol)**	**Melting Point (°C)**
DTBA	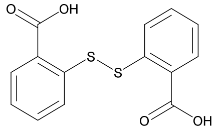 2,2′-Dithiodibenzoic acid	306,35	287-290
4-AFD	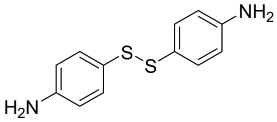 4-Aminophenyl disulfide	248.37	77-78
DTDA	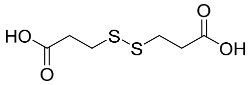 3,3′-Dithiodipropionic acid	210,27	155-158
IM	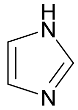 Imidazole	68,07	88-91

Table S2. Acronyms and composition of formulations ECMO/DTBA$_x$DTDA$_y$ - DTBAx 4-AFD$_y$.

Formulations	Composition (% in mol)		mol			Mixing ratio of ELO: DTBA/DTDA	IM (wt.%)
	DTBA	DTDA	ECMO	DTBA	DTDA		
ECMO/DTBA$_{100}$	100	0	$1.12\cdot10^{-3}$	$5.61\cdot10^{-4}$	/	1:1	1
ECMO/DTBA$_{70}$DTDA$_{30}$	70	30	$1.12\cdot10^{-3}$	$3.93\cdot10^{-4}$	$3.36\cdot10^{-4}$	1:1	1
ECMO/DTBA$_{50}$DTDA$_{50}$	50	50	$1.12\cdot10^{-3}$	$2.8\cdot10^{-4}$	$2.8\cdot10^{-4}$	1:1	1
ECMO/DTBA$_{30}$DTDA$_{70}$	30	70	$1.12\cdot10^{-3}$	$3.36\cdot10^{-4}$	$3.93\cdot10^{-4}$	1:1	1
ECMO/DTDA$_{100}$	0	100	$1.12\cdot10^{-3}$	/	$5.61\cdot10^{-4}$	1:1	1

Formulations	Composition (% in mol)		mol			Mixing ratio of ELO: DTBA/4-AFD	IM (wt.%)
	DTBA	4-AFD	ECMO	DTBA	4-AFD		
ECMO/DTBA$_{70}$4-AFD$_{30}$	70	30	$1.12\cdot10^{-3}$	$3.93\cdot10^{-4}$	$3.36\cdot10^{-4}$	1:1	1
ECMO/DTBA$_{50}$4-AFD$_{50}$	50	50	$1.12\cdot10^{-3}$	$2.8\cdot10^{-4}$	$2.8\cdot10^{-4}$	1:1	1
ECMO/DTBA$_{30}$4-AFD$_{70}$	30	70	$1.12\cdot10^{-3}$	$3.36\cdot10^{-4}$	$3.93\cdot10^{-4}$	1:1	1
ECMO/4-AFD$_{100}$	0	100	$1.12\cdot10^{-3}$	/	$5.61\cdot10^{-4}$	1:1	1

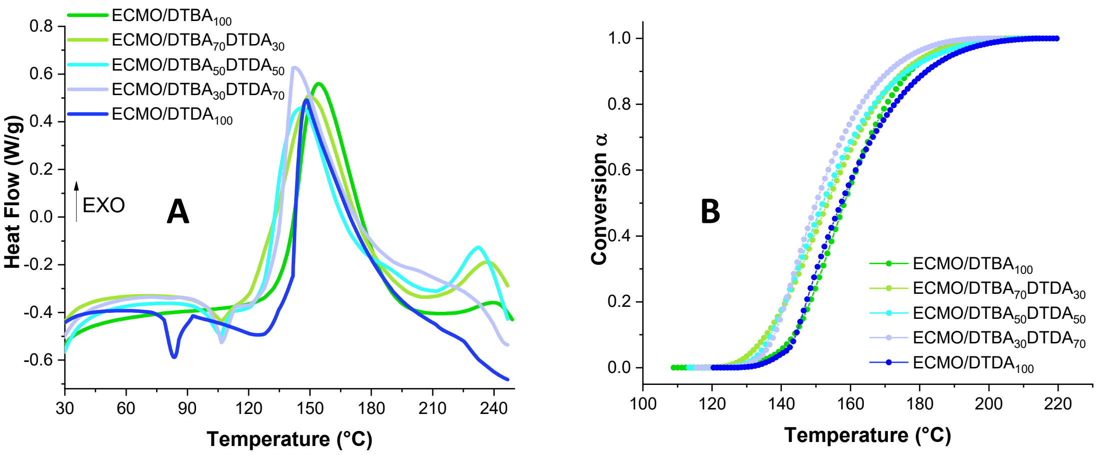

Figure S1. - DSC thermograms and degree of conversion α (B) for the ECMO bio-epoxy monomers combined with the diacids aromatic/aliphatic hardeners in R 1:1 and 1 wt.% of IM.

Table S3. DSC results for the ECMO/$DTBA_xDTDA_y$ systems.

Materials designation	T_{on} (°C)	T_{peak} (°C)	T_{end} (°C)	ΔH (J/g)
ECMO/$DTBA_{100}$	137	155	187	171
ECMO/$DTBA_{70}DTDA_{30}$	126	150	182	168
ECMO/$DTBA_{50}DTDA_{50}$	128	145	173	140
ECMO/$DTBA_{30}DTDA_{70}$	135	143	175	156
ECMO/$DTDA_{100}$	141	147	180	153

Table S4. Curing and post-curing protocol for the ECMO/$DTBA_xDTDA_y$ and ECMO/$DTBA_x4AFD_y$systems.

Materials designation	Curing	Post-curing	Aspect
ECMO/$DTBA_{100}$	130 °C – 60 min	170 °C – 30 min	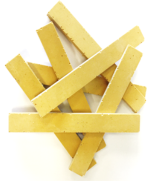
ECMO/$DTBA_{70}DTDA_{30}$	130 °C – 60 min	170 °C – 30 min	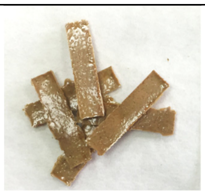
ECMO/$DTBA_{50}DTDA_{50}$	130 °C – 60 min	170 °C – 30 min	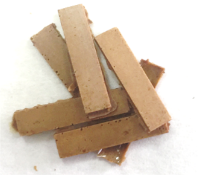
ECMO/$DTBA_{30}DTDA_{70}$	130 °C – 60 min	170 °C – 30 min	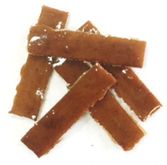
ECMO/$DTDA_{100}$	140 °C – 60 min	180 °C – 30 min	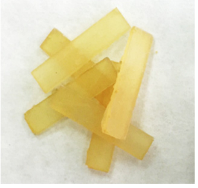
ECMO/$DTBA_{70}$4-AFD_{30}	120 °C – 60 min	160 °C – 30 min	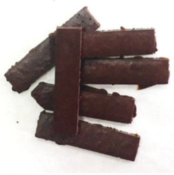
ECMO/$DTBA_{50}$4-AFD_{50}	20 °C – 60 min	160 °C – 30 min	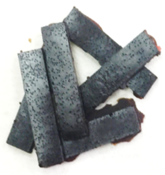

ECMO/DTBA$_{30}$4-AFD$_{70}$	120 °C – 60 min	160 °C – 30 min	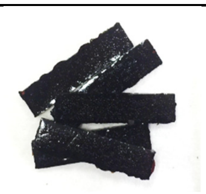
ECMO/4-AFD$_{100}$	180 °C – 150 min	200 °C – 30 min	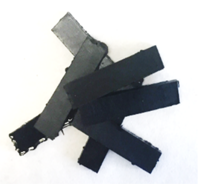

Table S5. Reprocessing conditions and aspects of the reprocessed ECMO/DTBA$_x$DTDA$_y$ and ECMO/DTBA$_x$4AFD$_y$ thermosets.

Materials	Reprocessing conditions	Aspect	Materials	Reprocessing conditions	Aspect
ECMO/ DTBA$_{100}$	170 °C 10 min 2 Tons	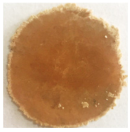			
ECMO/ DTBA$_{70}$DTDA$_{30}$	150 °C 15 min 1 Ton	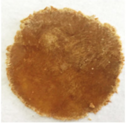	ECMO/ DTBA$_{70}$ 4-AFD$_{30}$	150 °C 10 min 1 Ton	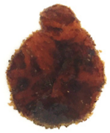
ECMO/ DTBA$_{50}$DTDA$_{50}$	150 °C 10 min 1 Ton	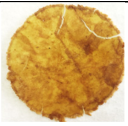	ECMO/ DTBA$_{50}$ 4-AFD$_{50}$	150 °C 10 min 0.5 Tons	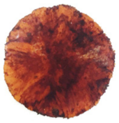
ECMO/ DTBA$_{30}$DTDA$_{70}$	150 °C 10 minutes 1 Ton	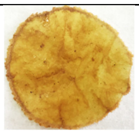	ECMO/ DTBA$_{30}$ 4-AFD$_{70}$	150 °C 10 min 0.5 Tons	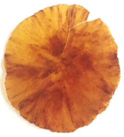
ECMO/ DTDA$_{100}$	170 °C 10 minutes 1 Ton	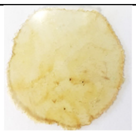	ECMO/ 4-AFD$_{100}$	150 °C 10 min 0.3 Tons	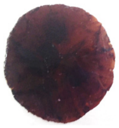

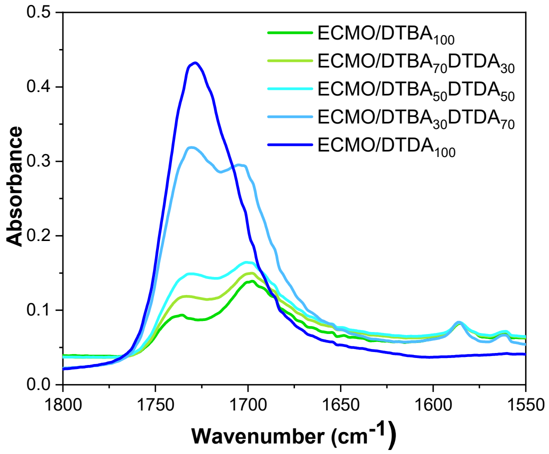

Figure S2. - FT-IR zoom of the ECMO/DTBAxDTDAy blends in the region from 1800 to 1150 cm-1.

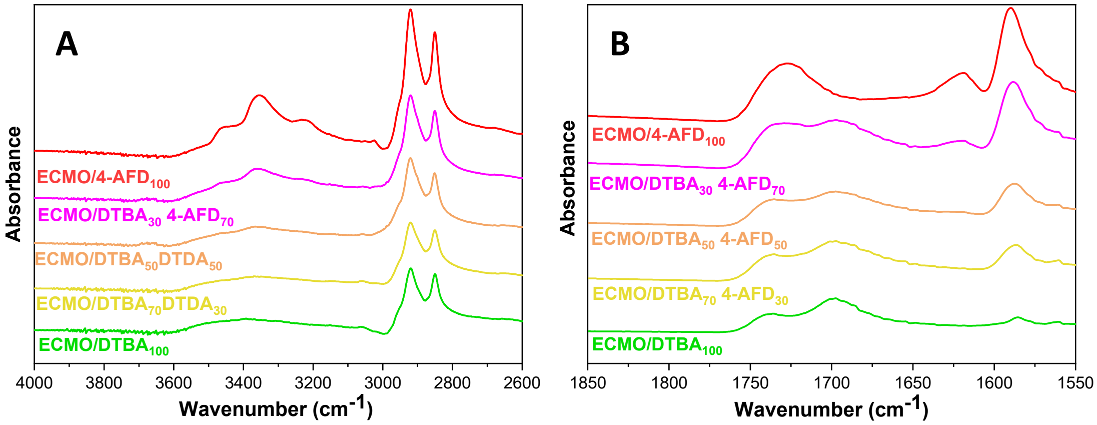

Figure S3. Evolution of FT-IR spectra of the uncured ECMO/DTBAx4-AFDy blends: **(A)** zoom in the region of 4000-2600 cm-1 and **(B)** 1850-1550 cm-1.

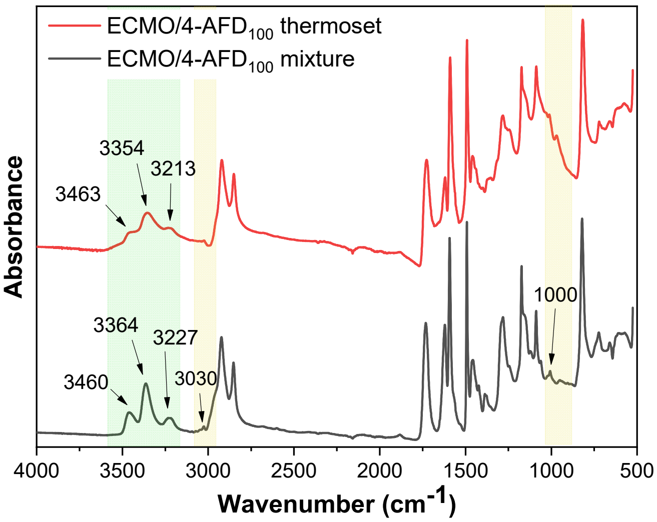

Figure S4. - FT-IR evolution of the reactive groups of the ECMO/4-AFD$_{100}$ in unreacted mixture and after the curing protocol.

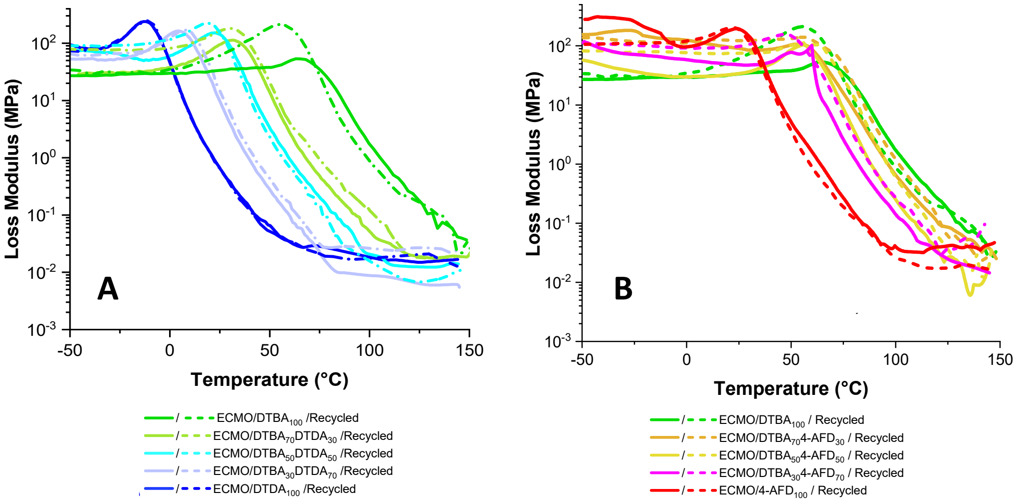

Figure S5. – Loss Modulus vs. temperature for the virgin and recycled **(A)** ECMO/DTBA$_x$DTDA$_y$ and **(B)** ECMO/DTBA$_x$4-AFD$_y$ thermosetting resins.

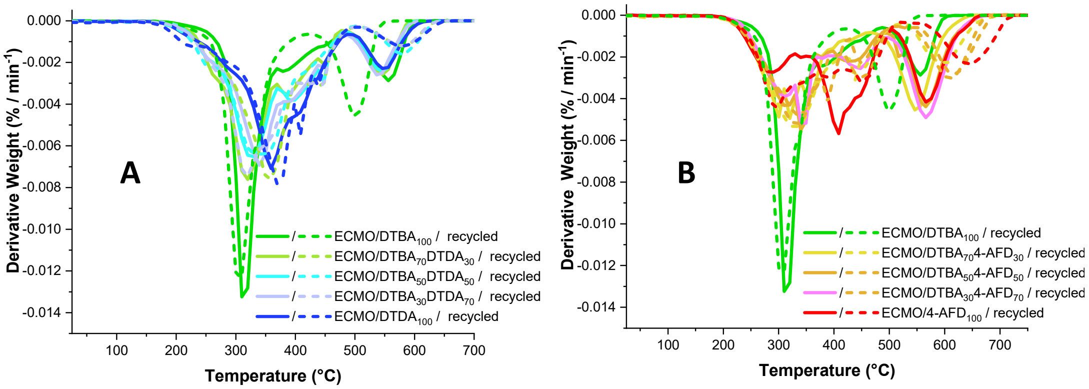

Figure S6. - DTG for the neat ECMO/DTBA$_x$DTDA$_y$ and combination of the diacids crosslinkers **(A)** and neat ECMO/DTBA$_x$4-AFD$_y$ and mixture of diacid/diamine blends **(B)**.

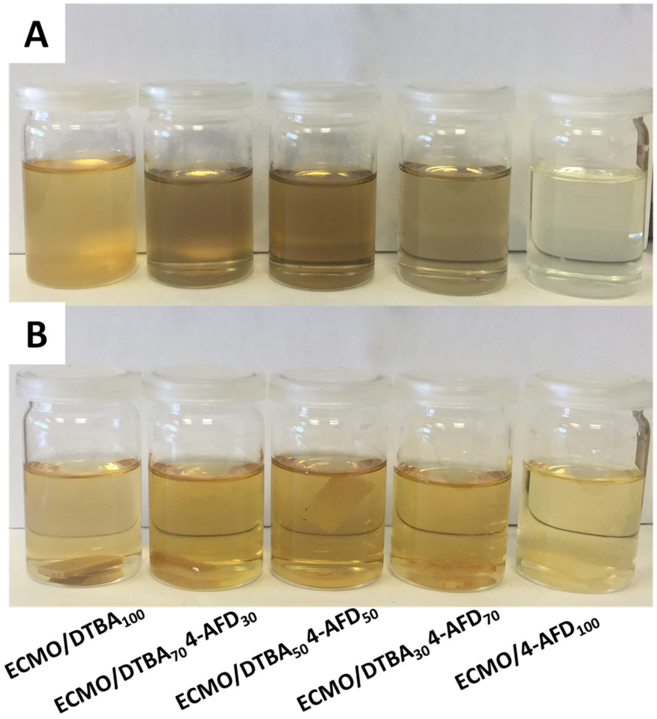

Figure S7. (A) Chemical recycling in DMF solution of dithiothreitol for ECMO/DTBA$_x$DTDA$_x$ after 24 hours at 50 °C and **(B)** solvent resistance in 1N NaOH solution after 3 days at room temperature.

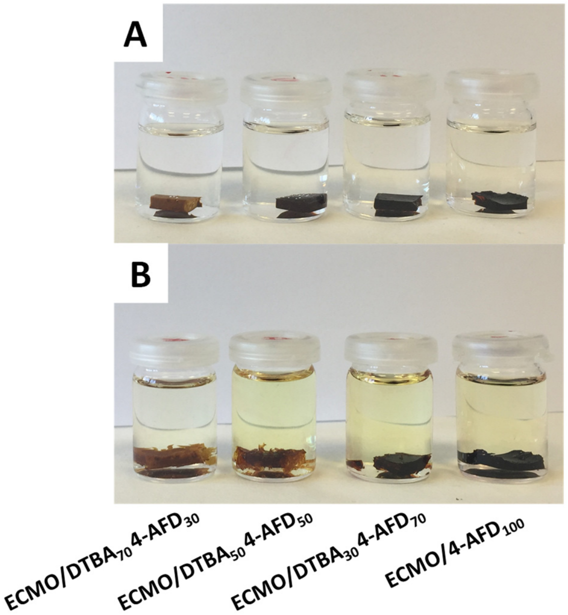

Figure S8. – Solvent resistance tests in **(A)** ethanol and **(B)** acetone after 72 hours at room temperature for the ECMO/DTBA$_x$4-AFD$_y$ resins.

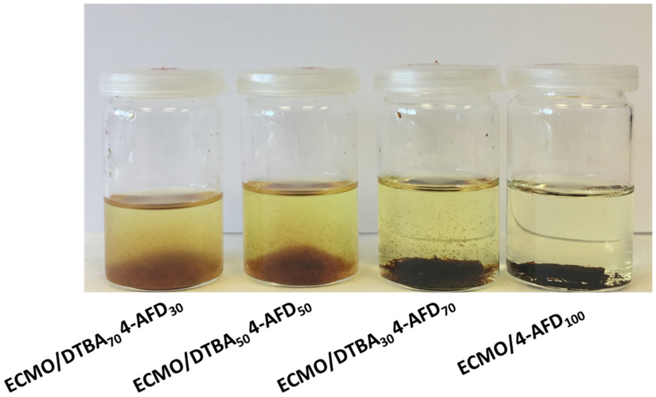

Figure S9. Solvent test in THF for ECMO/DTBA$_x$4-AFD$_y$ resins after 72 hours at room temperature.
